# Optimizing assessment of CD30 expression in Hodgkin lymphoma by controlling for low expression

**DOI:** 10.14670/HH-18-644

**Published:** 2023-06-21

**Authors:** Shoib Sarwar, Margaret E. Tome, Dean Billheimer, Catherine Spier, Catharine L Smith, Daniel Persky, Monika Schmelz

**Affiliations:** 1 Department of Pathology, University of Arizona, Tucson, AZ, USA; 2 Department of Pharmacology, University of Arizona, Tucson, AZ, USA; 3 Department of Epidemiology and Biostatistics, University of Arizona, Tucson, AZ, USA; 4 Pharmacology and Toxicology, College of Pharmacy, University of Arizona, Tucson, AZ, USA; 5 University of Arizona Cancer Center, Tucson, AZ, USA

**Keywords:** Hodgkin lymphoma, Reed-Sternberg cells, CD30, Immunohistochemistry, Membrane staining, Cytoplasmic punctate staining, Membrane trafficking

## Abstract

Since the approval of brentuximab vedotin (BV), assessment of CD30 status by immunohistochemistry gained increasing importance in the clinical management of patients diagnosed with CD30-expressing lymphomas, including classical Hodgkin lymphoma (CHL). Paradoxically, patients with low or no CD30 expression respond to BV. This discrepancy may be due to lack of standardization in CD30 staining methods. In this study, we examined 29 cases of CHL and 4 cases of nodular lymphocyte-predominant Hodgkin lymphoma (NLPHL) for CD30 expression using a staining protocol that was designed to detect low CD30 expression levels, and an evaluation system similar to the Allred scoring system used for breast cancer evaluation. For CHL, 10% of cases had low scores and 3% were CD30 negative, with 3 cases in which the majority of tumor cells showed very weak staining. Unexpectedly, one of four cases of NLPHL was positive. We demonstrate intra-patient heterogeneity in CD30 expression levels and staining patterns in tumor cells. Three CHL cases with weak staining may have been missed without the use of control tissue for low expression. Thus, standardization of CD30 immunohistochemical staining with use of known low-expressing controls may aid in proper CD30 assessment and subsequent therapeutic stratification of patients.

## Introduction

Hodgkin lymphoma (HL) is a neoplasm with an estimated 8500 newly diagnosed cases in the USA in 2018 with approximately 12% resulting in death (Hoppe et al., 2018). There are two types of HL, classical HL (CHL), which is more common and accounts for 95% of cases, and the less frequent nodular lymphocyte-predominant HL (NLPHL) (Hoppe et al., 2018). Typically, pathognomonic Reed-Sternberg cells (RSCs) in CHL express CD30 (Swerdlow et al., 2017), a cell membrane protein that is a member of the tumor necrosis factor receptor family (TNFRSF8). Brentuximab vedotin (BV, ADCETRIS^®^) is an antibody-drug conjugate (ADC) consisting of an anti-CD30 monoclonal antibody covalently linked to the microtubule-disrupting agent monomethyl auristatin E (MMAE) by a protease-cleavable linker (Francisco et al., 2003; Okeley et al., 2010; Senter and Sievers, 2012). Brentuximab vedotin is currently approved for CHL, anaplastic large cell lymphoma (ALCL) (another lymphoma where malignant cells uniformly express CD30), as well as CD30-expressing peripheral T-cell lymphomas and mycosis fungoides (MF) [ADCETRIS prescribing information].

While CD30 expression has been described as the critical immunophenotypic characteristic of RSCs, there are reports of heterogeneous expression and even CD30-negative Hodgkin lymphoma cases (Miettinen, 1992; von Wasielewski et al., 1997; Rüdiger et al., 1998a,b; Flangea et al., 2006; Salama et al., 2010; Ahmed et al., 2011). Furthermore, patients with low or no CD30 expression by immunohistochemical testing can respond to treatment (Horwitz et al., 2014; Kim et al., 2015; Bartlett et al., 2017). There are plausible biologic rationales for this. A study to measure binding properties and internalization kinetics of BV showed that binding of only a few BV molecules is required for clinical activity (Fromm et al., 2012). In addition, MMAE exerts a secondary effect by diffusing from the CD30 positive cells into their surrounding microenvironment where it causes immunogenic cell death. However, the discrepancy between CD30 expression and response to BV may be technical, due to a lack of standardization of CD30 immunostaining methods. Most studies on CD30 expression in CHL did not report intensity levels and percentages of positive tumor cells. There was even a lack of consistent definition of positive CD30 expression. Since CD30 is emerging as a predictive biomarker that guides treatment, guidelines for CD30 testing were recently established by an expert panel consensus (Gru et al., 2023). CD30 immunohistochemistry is required for all patients with suspected CHL for differential diagnostic considerations. The panel reinforced and summarized that immunohistochemistry is the preferred methodology and any degree of CD30 expression should be reported (Gru et al., 2023). Antigen expression levels and patterns are dynamic key parameters for efficient drug delivery, as they will determine how much ADC will bind to the tumor cell and be internalized (Bander, 2013).

Since we started our study prior to the publication of the CD30 testing guidelines, we followed the ASCO-CAP test guideline recommendations for evaluating Her2/neu expression in breast cancer. The objective of this study was to examine CD30 expression levels and staining patterns in an archival case series by using a cell array with known CD30 expression levels to control for the detection of low expression levels, and a scoring system without a cut-off threshold similar to the Allred scoring system (Allred et al., 1998).

## Materials and methods

### Patient tissues

Thirty-three formalin-fixed, paraffin-embedded samples from 33 patients diagnosed with Hodgkin Lymphoma were acquired through the Arizona Lymphoid Tissue and Blood Repository (ALTBR) at the University of Arizona. Histologic and diagnostic types were obtained from the pathology reports (age, sex, location and type of tissue, and some immunohistochemistry (IHC) stain results from time of diagnosis). For some cases, but not all, the stage and information on the presence or absence of extra nodal disease was available (Table 1). The University of Arizona Institutional Review Board in accordance with the Declaration of Helsinki approved the use of these human tissues and clinical data.

### Cell culture and control tissue

To have standardized controls available with known CD30 expression levels, the following cell lines were used: cell line L-428, originated from a Hodgkin lymphoma showing high expression of CD30. TF-1a is a lymphoblast cell line that came from an erythroleukemia patient and has moderate expression levels of CD30. The MOLM-13 cell line originated from a patient with acute myeloid leukemia expressing low levels of CD30. Finally, HCT 116 is a human colon carcinoma that is CD30 negative.

L-428 and MOLM-13 were purchased from DSMZ (Deutsche Sammlung von Mikroorganismen und Zellkulturen) while cell lines TF-1a and HCT116 were obtained from ATCC (American Type Culture Collection). Upon receipt, all were subjected to authentication analysis. For creating tissue microarrays, the cell lines were cultured at Seattle Genetics, Inc headquarters. For protein analysis by western blotting, the cell lines were cultured in our laboratory. Cell lines L-428, TF-1a, and MOLM-13 were cultured in RPMI 1640 containing 10% FBS at densities between 2x10^5^ and 2x10^6^ cells/ml. HCT116 cells were cultured in DMEM containing 10% FBS (Thermo Fisher Scientific, Waltham, MA).

For the creation of tissue microarrays, cell pellets of the four genetically unaltered cell lines, as described above, were processed and embedded in paraffin blocks by scientists at Seattle Genetics, Inc., gene copies were compared to IHC data, and batches of microarray slides were prepared, tested, and validated for CD30 expression levels by IHC at Seattle Genetics Inc. headquarters prior to being shipped to our laboratory.

In brief, cells were cultured there in vendor recommended media as described above. Adherent cells (HCT116) were collected using ATCC cell dissociation solution at 80-90% confluency. Suspension cells were collected when they reached 1x10^6^ cell/ml density or at the density recommended by supplier for splitting. Per pellet 5-10x10^6^ cells were used. Cells were washed twice with cold PBS and fixed with 10% neutral-pH phosphate buffered formalin for 12-24 hours followed by embedding the cell pellets in HistoGel (VWR cat #83009-992) according to manufacture instructions. HistoGel-embedded cells were placed in 70% Ethanol prior to processing and embedding in paraffin block. For creating tissue arrays, the paraffin blocks with the four cell lines were send to Tissue Array (TissueArray.com LLC, Derwood, MD).

### Protein extract preparation, Western blotting

Whole cell lysates for Western blotting were prepared as follows. Briefly, 2-5x10^6^ cells were pelleted by centrifugation and washed once with ice-cold PBS containing protease inhibitors (Roche Complete). Cells were resuspended in up to 150 μl of RIPA buffer (50 mM Tris pH 8.0, 150 mM NaCl, 1% Triton X-100, 0.5% sodium deoxycholate, 0.1% SDS, Roche cOmplete^™^ protease inhibitors) and phosphatase inhibitors (10 mM sodium fluoride, 25 mM beta-glycerophosphate, 0.1 mM sodium orthovanadate). All reagents were purchased from Sigma-Aldrich (St. Louis, MO). Resuspended cells were sonicated on high for 15 seconds in a Bioruptor water bath sonicator and then passed through Qiashredders (Qiagen, Germantown, MD) by microcentrifugation (5 min, 4°C, 18000xg). The supernatant (whole cell extract) was transferred to clean tubes. Protein concentrations were determined by Bradford assay.

Extracted proteins were separated by SDS polyacrylamide gel electrophoresis using 8% resolving gels. Protein was then transferred to nitrocellulose membranes by Western Transfer (2h, 4°C, 400 mA). Immunoblotting was performed with primary antibodies to CD-30 (clone BerH2, Santa Cruz Biotechnology, Dallas, TX) and GAPDH (Santa Cruz Biotechnology) and HRP-conjugated goat anti-mouse secondary antibody (Jackson Immunoresearch). Antibody-bound proteins were detected by chemiluminescent imaging using a Chemidoc instrument (BioRad Laboratories, Hercules, CA).

These experiments were carried out in triplicate by subjecting three individually grown batches of the cell lines to western blotting.

### Immunohistochemistry (IHC)

All Hodgkin’s cases in this study were stained with the mouse monoclonal antibody Confirm CD30 (clone BerH2), a Ready-To-Use formula (Ventana Medical Systems, Tucson, AZ) using IHC protocol 1, as described below. Later, Ventana replaced the Confirm CD30 (BerH2) with a product called just CD30, also a Ready-To-Use antibody (clone BerH2) against CD30 antigen, but with a proprietary formulation different from the CONFIRM CD30 formula. Lot numbers G00161 and G03198 of these newer formulas were tested. In addition, concentrated mouse monoclonal antibodies, all clone BerH2, from two vendors (DakoCytomation, Carpinteria, CA and Santa Cruz Biotechnology, Dallas, TX) were evaluated at dilutions of 1:20, 1:40, 1:100 and 1:200. The CD30 antibody clone CON6D/C2 from Thermo Fisher Scientific, Waltham, MA) was also tested at dilutions of 1:200, 1:1000, and 1:2000. All concentrated antibodies were diluted in Antibody Diluent (Ventana Medical Inc.) All immunostainings were performed on a Benchmark XT fully automated system (Ventana Medical Systems).

### IHC Protocol 1

IHC was performed on 3-5μm thick sections. All secondary reagents were purchased from Ventana Medical Systems (Tucson, AZ). In brief, after automated online deparaffinization of the tissue, HIER (heat-induced epitope retrieval) antigen retrieval was automatically performed on the stainer with pre-set temperature conditions using Ventana’s Tris-based cell conditioning 1 solution (CC1, pH 8.5) for 64 min, followed by blocking endogenous peroxidase for 4 min. The primary antibody was incubated for 56 min with heat applied (37°C) For antigen detection, the biotin-free OptiView DAB Kit was used. The OptiView DAB Kit components were incubated as follows: The Optiview HQ Universal Linker for 12 min, the Optiview HRP Multimer for 12 min, the Optiview H202-Optiview DAB mix for 8 min, and the Optiview Copper for 4 min. The OptiView Amplification Kit (Optiview Amplification H_2_O_2_ & Optiview Amplification Multimer) was used with a 4 min incubation time for each component. The latter was needed for the detection of low CD30 expression in MOLM-13 cells in conjunction with the Ventana Confirm CD30/Ready-To-Use CD30 antibody. Hematoxylin II (4 min) and bluing reagent (4 min) were used for counterstaining.

### IHC protocol 2

IHC protocol 2 was identical to protocol 1 except for the application of heat for the primary antibody incubation, the time of primary antibody incubation, and the time of HIER antigen retrieval. Hier antigen retrieval was performed for 72 min, and the primary antibody was incubated for 1 hour at room temperature with no heat applied.

### Scoring

An Olympus BX-41 microscope equipped with an Olympus DP-21 camera and integral image acquisition software was used for viewing slides and capturing images. First, hematoxylin & eosin (H&E) stained sections were viewed to identify areas containing tumor cells. This was followed by a semi-quantitative analysis and scoring of CD30 staining in the same areas on sequential sections. A case was considered positive when the sample showed any number of RSCs with membrane staining or cytoplasmic staining. No minimum number of positive RSCs was determined as a threshold for CD30 positivity. IHC scoring was performed on a scale of 0 to 3+ for intensity (no staining=0, weak staining=1+, moderate staining=2+, strong staining =3+) and 0 to 4 for percent positive RSCs (0 indicates no stain; 0.5=<5%; 1=5%-25%; 2= 25%-50%; 3=50%-75%; and 4=75%-100%). For each case, scoring was performed in 3 randomly selected 60 x fields containing tumor cells followed by averaging the scores. An average of 100 tumor cells per case or sample were counted, and each cell was scored as negative or positive. The positive cells received scores for intensity levels of expression. The numbers of cells scored as positive were divided by the total cells counted and multiplied by 100 for a percentage positive score. As previously described for examining MHCII protein expression levels in diffuse large B-cell lymphoma (Wilkinson et al., 2012), CD30 expression scores using chromogenic IHC were calculated by adding average intensity value plus average value for percent positive cells. Thus, calculated overall scores had a possible range of expression of 0 to 7. To account for variation of intensity within the same field, the number of cells with 1+, 2+ and 3+ expression levels were computed. The level expressed by the highest percentage of cells was given to that field. For example, when in one field 58% of tumor cells expressed 3+, 21% 2+, and 13% 1+, the field was scored with a 3+. When the 3 fields per case had different intensity levels, the average among the 3 fields was calculated for the overall score. To assess the intra-patient variability, the percentages of cells for each intensity level per case were documented in addition to the overall score (Tables 1, 4).

Qualitative notation of membrane (Me) versus cytoplasmic/punctate (CP) staining of CD30 was made. Cytoplasmic “punctate” refers to either a single globular stain accumulation adjacent to the nucleus or to multiple smaller, punctate staining patterns throughout the cytoplasm in the absence of membrane staining. Two observers (SS and MS) independently performed scoring and the results were compared with an initial agreement of 31/33 (94% concordance). For the discordant cases, discussion and resolution were performed at a multiheaded microscope and the consensus result used in further analysis.

### Statistical analysis

Quantitative values were summarized using the mean and standard deviation, while count values were tabulated and described as percentages. Comparison of the proportion of positive-staining cases was conducted using a Pearson chi-square test, and secondarily using a Bayesian analysis with a beta-binomial model with uniform prior distributions for the unknown binomial parameters. In small sample sizes, the Bayesian test has been shown to have greater sensitivity than classical procedures (Brown et al., 2001).

## Results

### CD30 expression levels in the cell line controls

**Fig. 1.**
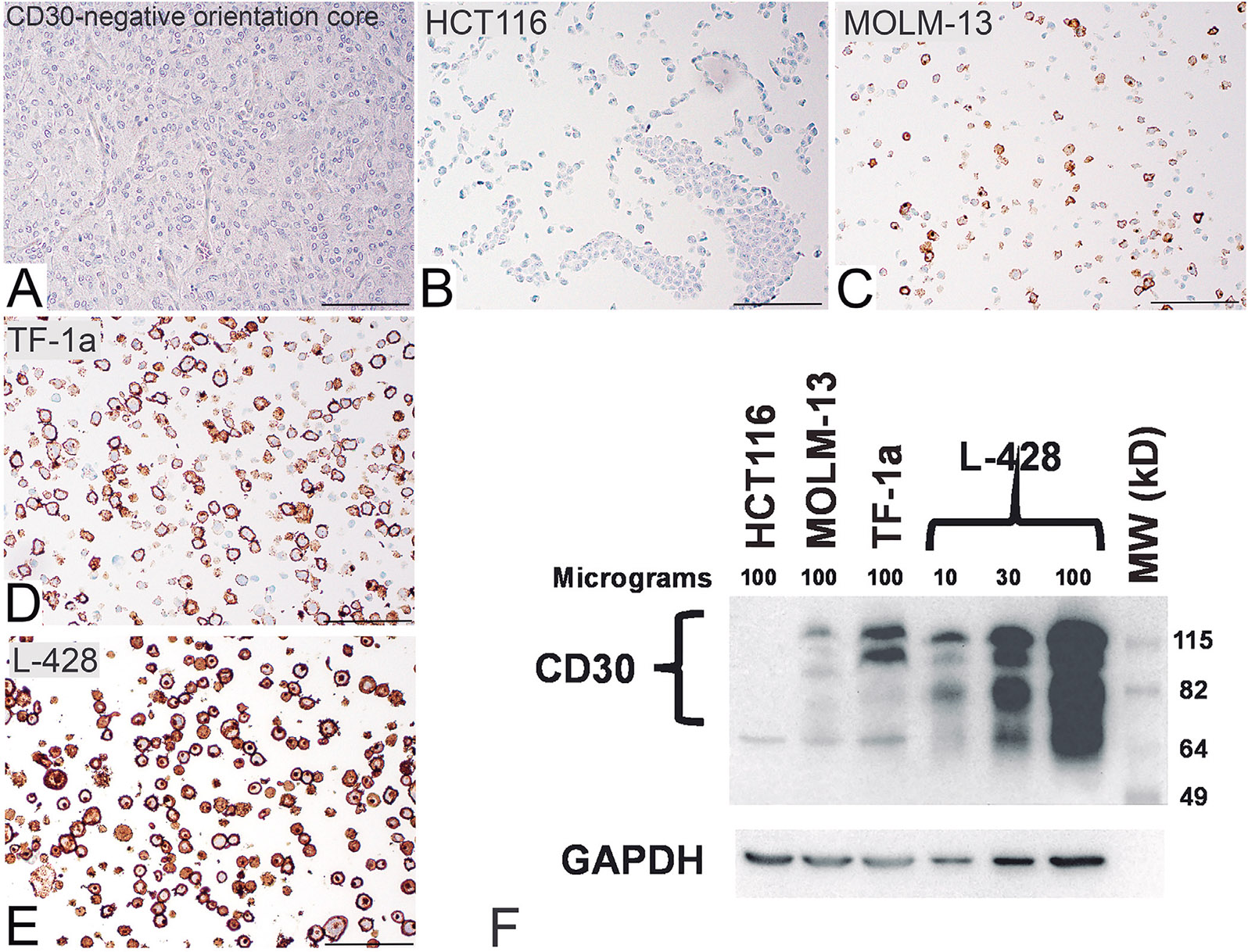
The anti-CONFIRM CD30 (Ventana Medical) using IHC protocol 1 shows staining in cells with low CD30 amounts in a tissue array constructed from various cell lines. **A.** CD30 negative orientation tissue core. **B.** HCT 116, a human colon carcinoma, which is negative for CD30. **C.** MOLM-13, originating from a patient with acute myeloid leukemia, which shows low expression levels of CD30. **D.** TF-1a, lymphoblasts from a patient with erythroleukemia, that shows moderate expression levels of CD30. **E.** L-428, originating from a Hodgkin lymphoma, which shows high expression of CD30. Panel **F** shows a Western blot carried out with CD30 antibody (clone BerH2) using whole cell extracts prepared from the same cell lines shown in panels **B-E**). The amount of extract loaded into each lane is indicated. GAPDH was also blotted to serve as a loading control. Scale bars: 100 *μ*m.

Since low expression of CD30 was shown to be clinically relevant, a staining protocol was developed so that the high, medium, and low CD30-expressing and - negative cells were correspondingly stained (Fig. 1A-E). The challenge was to detect CD30 in the low-expressing MOLM13 cells, which was achieved with the CD30 CONFIRM antibody from Ventana Medical using IHC protocol 1 (See Methods and Materials).

As a second approach to confirming the relative levels of CD30 in the cell lines we carried out Western Blotting with the CD30 clone BerH2 antibody using whole cell extracts from L-428, TF-1a, MOLM-13, and HCT116 cells. As shown in Figure 1F, each lane was loaded with 100 μg of protein from HCT116, MOLM-13, and TF-1a. Because L-428 cells have very high expression of CD30, we included lanes loaded with 10, 30, and 100 μg protein for comparison purposes. The results confirm the relative levels of CD30 expression noted in the IHC analysis (Fig. 1A-E) with L-428 having the most CD30 expression and MOLM-13 having the least. The negative control cell line, HCT116, had no detectable level of CD30.

### Optimization of CD30 staining in the cell line microarray

**Fig. 2.**
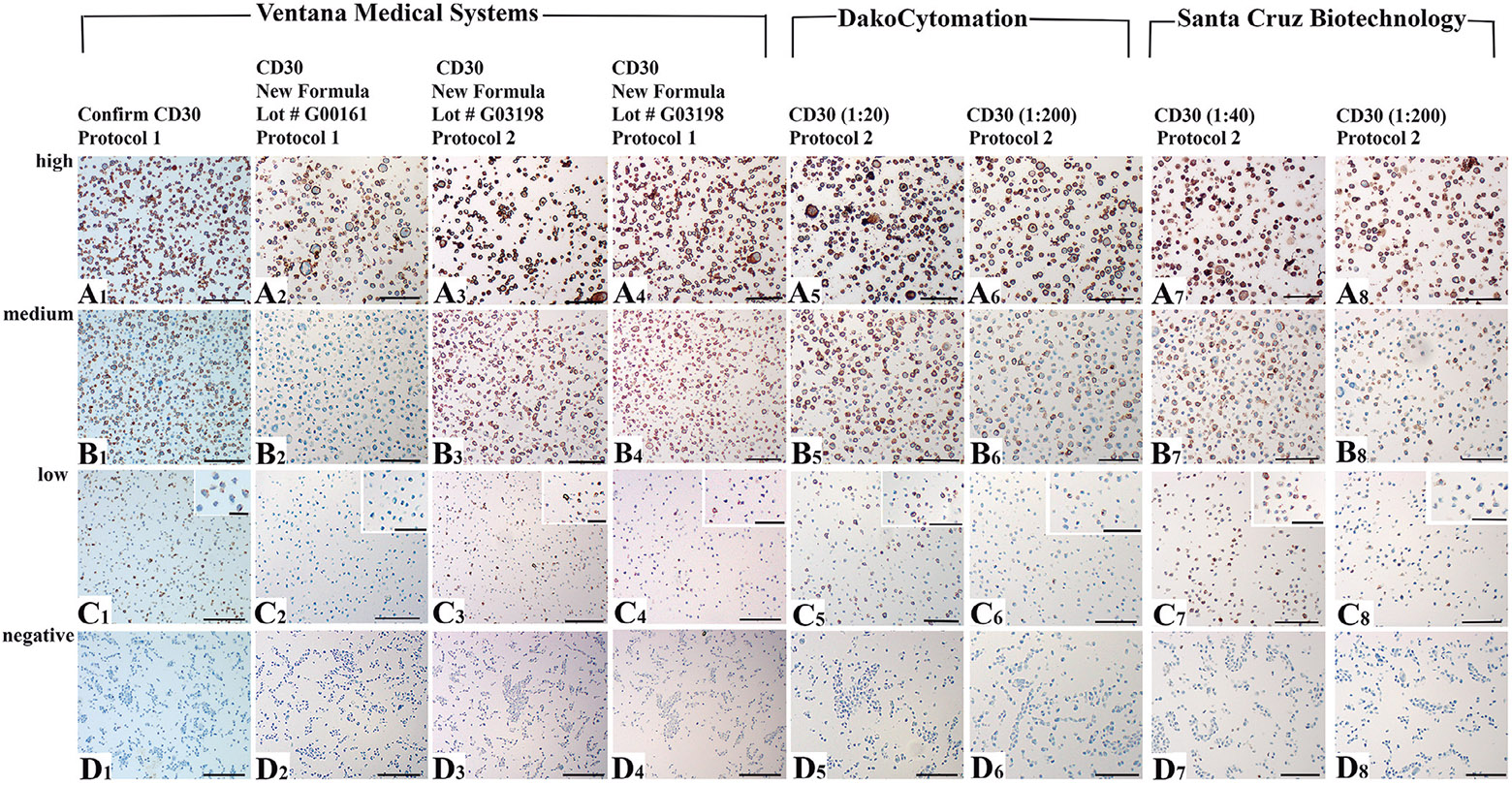
Comparison of various CD30 antibodies from different vendors (all clone BerH2) and IHC protocols on the control tissue array. **A1-A8.** L-428 cells (high CD30 expressing) show somewhat comparable staining. **B1-B8.** TF-1a cells (moderate CD30 expressing). **C1-C8.** MOLM-13 (low CD30 expressing). **D1-D8.** HCT 116 cells (CD30 negative). In the high CD30 expressing cells **(A1-A8)** the different antibodies, dilutions and protocols were somewhat comparable. However, in the moderately CD30 expressing cells, staining differences between antibodies, lot numbers, dilutions and protocols become obvious, and are significant in the low CD30 expressing cells. The newer Ventana CD30 formula Lot# G00161 lost signal in the moderately expressing cells **(B2)** and did not detect CD30 staining in the low expressing cells when using IHC protocol 1 **(C2)** as compared to the Confirm CD30 antibody **(B1, C1)**. For the newer formula of Ventana CD30 antibody Lot # G03198, protocol 2 works better in detecting CD30 in the low expressing cells **(C3)** as compared to protocol 1 **(C4)**. For the detecting CD30 in the low expressing cells, the 1:20 dilution of the concentrated antibody from DakoCytomation worked best in conjunction with protocol 2 **(C5)**, while the 1:40 dilution of the antibody from Santa Cruz Biotechnology worked best in conjunction with protocol 2 **(C7)**. Note that the CD30 negative controls showed no unspecific staining and no background with antibody dilutions and protocols that detect CD30 in the low expressing cells **(D1, D3, D5, D7)**. Scale bars: 100 *μ*m; inserts in C1-C8, 30 *μ*m.

The CD30 CONFIRM antibody used in the IHC analysis shown in Figure 1A-E was replaced by Ventana Medical with a newer Ready-to Use formulation of the CD30 clone BerH2 antibody. When this new formulation was used in IHC protocol 1, the CD30 signals in the low-expressing MOLM-13 cells were completely lost (Fig. 2C2), and significantly reduced in the intermediate-expressing TF-1a cells (Fig. 2B2). Only a different lot number of the new formula and a protocol change (protocol 2 - see Methods and Materials) produced the best result in detecting CD30 expression in MOLM-13 cells (Fig. 2B3,C3); protocol 1 with the same lot number produced a significantly weaker CD30 staining in MOLM-13 cells (Fig. 2C4). We also tested concentrated CD30 antibody formulations, all clone BerH2, from different vendors. Using protocol 2, the best results for detection of CD30 in MOLM-13 cells were obtained using the DakoCytomation antibody at dilution 1:20 (Fig. 2C5), or the Santa Cruz antibody at dilution 1:40 (Fig. 2C7). In contrast, the CD30 signal was very weak (Fig. 2C6,C8) when these same antibodies (Dako-Cytomation or Santa Cruz) were used at a 1:200 dilution. The antibody clone CON6D/C2 worked best in detecting CD30 in MOLM13 cells at a dilution 1:1000 using protocol 1. However, this antibody showed a high background in all cell lines including the CD30-negative HCT116 cells, which could not be decreased by protocol changes without losing detection of CD30 in MOLM-13 cells (data not shown). Interestingly, the two staining protocols and the various antibodies, lot numbers and dilutions produced a somewhat comparable strong staining in the L428 cells (Fig. 2A1-A8).

### Patient tissue

A total of 33 cases, 15 (45%) females and 18 (55%) males, were included in this study (Table 1). Mean age of all the patients was 34.7±18 (range 4 to 72 years). The case series consisted of 29 CHL and 4 NLPHL. We included NLPHL cases as an expected negative set of patients. The histological subtypes of CHL cases included 72% (21/29) nodular sclerosis, 14% (4/29) mixed cellularity, 10% (3/29) lymphocyte rich, and 3% (1/29) lymphocyte depleted subtype. Thirty-one samples were initial diagnostic specimens, and two samples were recurrent specimens from patients with a history of lymphoma. The initial diagnostic specimens of these two patients with recurrent lymphoma were not available.

### CD30 overall scores

**Fig. 3.**
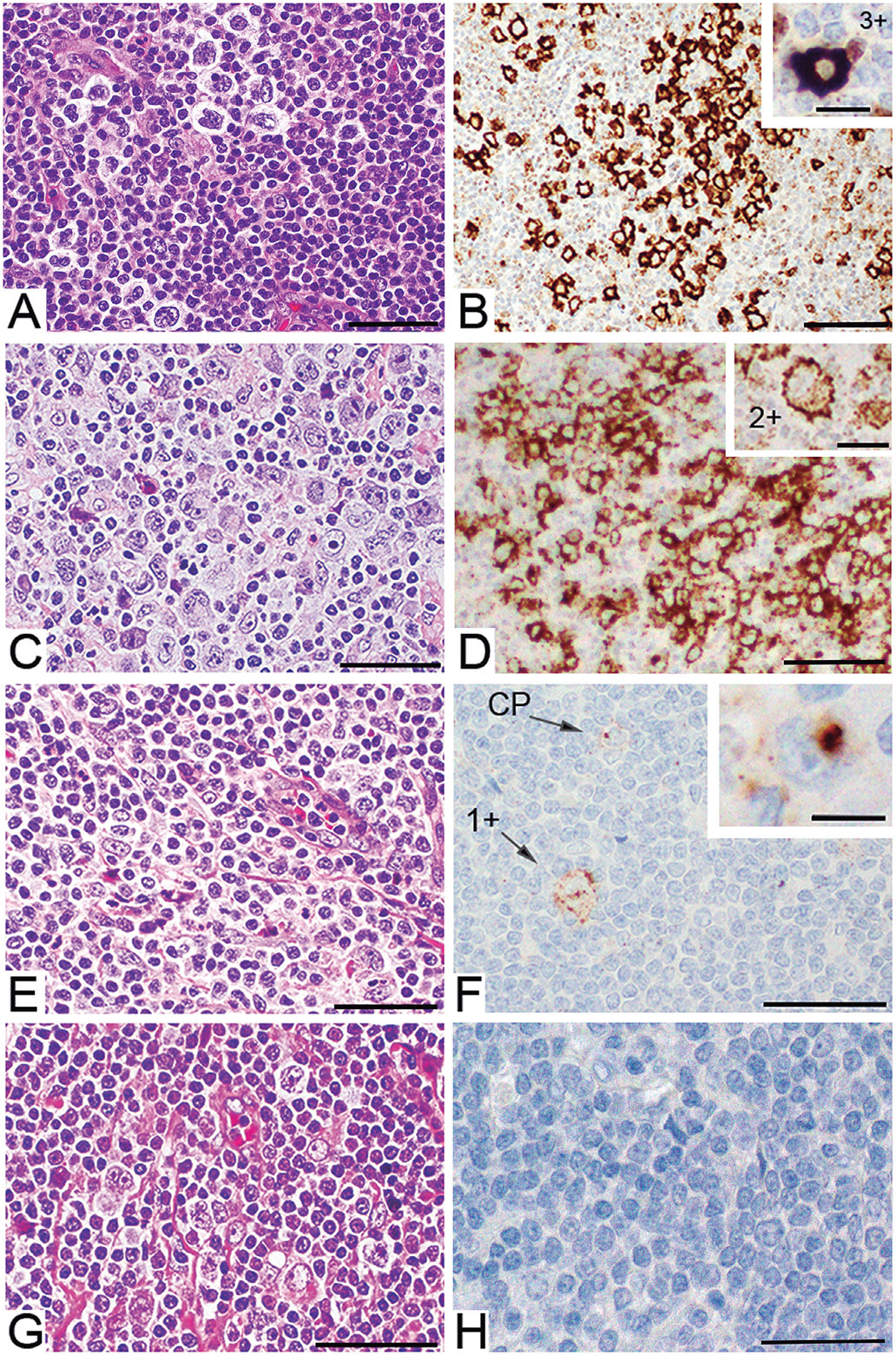
CD30 (BerH2) staining in Hodgkin lymphoma (HL) cases. **A, C, E, G.** Show H&E staining of representative tumor areas showing RSCs. **B, D, F, H.** Show CD30 staining controlled by the cell line array. **A, B.** Case 27 shows membrane staining with homogeneous high intensity levels. Some small number of T-lymphocytes showed a punctate staining pattern, which was interpreted as unspecific background staining. Insert in (B) shows a 3+ tumor cell. **C, D.** Case 24 shows membrane staining with heterogeneous intensity levels. Insert in (D) shows a tumor cell with a 2+ staining intensity. **E, F.** Case 8 shows a cytoplasmic punctate, and often perinuclear pattern in the absence of membrane staining (CP). The insert in (F) shows the cytoplasmic pattern in a tumor cell at higher magnification. **G, H.** Case 33 was negative for CD30. Scale bars: A-H, 50 *μ*m; inserts in B, D, F, 30 *μ*m.

All cases in this study were immunostained with the CD30 CONFIRM antibody (clone BerH2) from Ventana using IHC protocol 1. Clone BerH2 was chosen because it is the most frequently used clone in diagnostic testing. The mean overall CD30 score for classical HL was 5.5 + 1.74 (n=29), and 0.5 + 0.86 (n=4) for NLPHL. The frequency distribution of CD30 expression based on the overall scores and the staining patterns is given in Table 2. In one case (3%) of CHL and in three cases (75%) of NLPHL, the tumor cells showed complete absence of CD30 staining (Fig. 3G,H), whereas 28 cases of CHL (97%) and one case (25%) of NLPHL expressed CD30 to various degrees (p=0.01; Bayesian probability that CHL exhibits greater expression=0.99). The overall score for CD30 positivity ranged from 0-7 for CHL and was 4 for the positive NLPHL case (Tables 1, 2). In CHL, most cases (66%) received high scores followed by medium scores in 21% and low overall scores in 10% of the samples (Tables 2, 3). In 10% of the CHL cases (3/29, Table 4), high percentages of the tumor cells showed very weak staining levels (1+) that may have been missed without the use of controls for low expression and by using a scoring cut-off.

In NLPHL, the one positive case received a medium score (Table 2). This was paradoxical, since NLPHL is described as mostly CD30 negative. Among CHL, the nodular sclerosis HL subtype exhibited high CD30 expression (score 6-7, 15/21, 71%) while a lower percentage of mixed, lymphocyte rich and depleted subtypes (3/8, 38%) exhibited high expression (p=0.10; Bayesian probability that nodular sclerosis has greater expression=0.95). While the difference did not reach statistical significance, these data suggest that CD30 expression may be greater in the nodular sclerosis subtype. A study with a larger number of cases could address this issue.

### Intra-patient target antigen heterogeneity

Intra-patient heterogeneity of CD30 expression was assessed by percent of RSCs that were either CD30 positive or negative within one case, staining intensity levels and staining patterns.

Intra-patient heterogeneity, or homogeneity respectively, of CD30 expression is summarized in Tables 1, 3 and 4.

In 17% (5/29) of CHL cases, CD30 was expressed on the membranes of all tumor cells. However, in 80% (23/29) of the CHL cases and in the one positive NLPHL case, the tumor mass encompassed tumor cells that were membrane positive with varying intensity levels and completely negative tumor cells (Tables 1, 3).

In CHL, two staining patterns at varying intensity levels were observed in RSCs: 1) membrane staining with or without perinuclear staining (Fig. 3B,D respectively), and 2) cytoplasmic staining in the absence of membrane staining (Fig. 3F). The intracellular, cytoplasmic staining appeared mostly as one large, globular stain accumulation adjacent to the nucleus (Fig. 2F), and occasionally as smaller dots throughout the cytoplasm. This cytoplasmic staining pattern was predominantly observed in cases with low CD30 expression.

In 27% (8/29) of CHL, both staining patterns between tumor cells within the same case were observed, membrane staining and cytoplasmic staining in the absence of membrane staining (Tables 1, 2, Fig. 3F). In 7% (2/29) of CHL, only the cytoplasmic staining pattern in the absence of any membrane staining was observed (Tables 1, 2). The cytoplasmic staining pattern was not observed in the one positive NLPHL case. In addition, in 45% (13/29) of CHL cases, the tumor cells showed heterogeneity of CD30 expression levels to various degrees (detailed in Table 4). Tumor cells with 1+, 2+ or 3+ staining levels often were admixed. The one NLPHL case that was CD30 positive also showed heterogeneity in expression levels (Table 4).

### CD30 staining in the tumor microenvironment

The tumor microenvironment was composed of small lymphocytes, mostly T-cells, eosinophils, plasma cells and occasionally histiocytes. In four cases, CD30 expression was observed on the membranes of very few small lymphocytes (data not shown). Otherwise, the membranes of most infiltrating cells were negative. In some cases, punctate nuclear CD30 staining was observed in small T-lymphocytes, which was interpreted as non-specific. In the NLPHL case, in which the tumor cells were CD30 positive, some CD30 positive immunoblasts were present but were excluded from the quantification (data not shown).

## Discussion

Although HL is now generally considered to have a favorable outcome with new modern therapeutic strategies, it remains important for pathologists and oncologists to establish reliable prognostic and therapeutic factors. The response to treatment in patients whose tumors expresses low CD30 levels makes the consistent identification of low expressing tumors important (Horwitz et al., 2014; Kim et al., 2015; Bartlett et al., 2017). Therefore, using controls with low levels of CD30 expression is of clinical relevance (Gru et al., 2023). To our knowledge, no study has used a tissue microarray with varying, known CD30 expression levels, ranging from zero to high, for optimization of the staining procedure, and a scoring system similar to the Allred system. In previous studies of CHL, the percentage of cases in which CD30 positivity in RSCs was reported ranged from 27% to 100% with 0 to 71% of cases lacking CD30 expression (Miettinen, 1992; Von Wasielewski et al., 1997; Rüdiger et al., 1998b; Watanabe et al., 2000; Salama et al., 2010; Ahmed et al., 2011; Paszkiewicz-Kozik et al., 2013).

In our series, we found 97% of the cases were positive for CD30, which falls into the upper percentage range of previous reports; this is consistent with modern diagnosis of CHL. However, many reports did not distinguish between membrane and/or cytoplasmic staining and did not report the estimated percentage of tumor cells that were CD30 positive in more detail. Most studies did not include very detailed comments on the focal expression of CD30 and only a few mentioned that not all tumor cells were positive in some cases. These distinctions may have therapeutic implications.

This study demonstrates the variability and complexity of CD30 expression in human tumors. Although we were not able to compare the highly sensitive staining protocol with other, less stringent staining protocols due to limited availability of tissue in this case series, we identified three cases with very low CD30 expression levels that may have been found negative with a less sensitive staining protocol. However, no treatment data were available on these patients.

The largest case series reported so far examined 1751 cases of CHL and found only 1.6% of cases to be CD30-negative (von Wasielewski et al., 1997). However, this study used a 20% cut-off for positivity, which may not be good practice considering the positive response of low CD30 expressing patients to BV treatment. Modern clinical trials such as the ECHELON-2 and ALCANZA trials for PTCL and CTCL respectively, used minimum of 10% of CD30 staining for eligibility (Kim et al., 2018; Jagadeesh et al., 2019). Although the variation in CD30 expression seen across studies could be explained to some extent by inter-patient variability, these differences may also be due to the lack of standardized immunostaining procedures and evaluation of staining.

The levels of CD30 expression showed a persistent trend toward higher expression in the nodular sclerosis subtype than in the other histological subtypes of CHL. Along with Schwab et al. (1982), who discovered CD30 expression in RSCs, other studies reported variation in intensity levels that had no correlation with histological type (Schwab et al., 1982; von Wasielewski et al., 2003; Flangea et al., 2006). Although statistical analysis did not achieve significance, this finding could be clinically relevant and needs further investigation in larger cohorts.

NLPHL is characterized by the lack of CD30 expression (Roberts et al., 2002). A small proportion of NLPHL cases have been reported to express CD30 (Nicholas et al., 1990; Miettinen, 1992; Rüdiger et al., 1998b; Anagnostopoulos et al., 2000; Ranjan and Naresh, 2003). The fact that we found 25% of the NLPHL cases to be CD30-positive may be related to the higher sensitivity of our staining method but could also be an anomaly due to a small sample size. If corroborated in a larger cohort, it could indicate a role for BV in this disease.

CD30 expression on the cell membrane is a key and dynamic parameter for binding of BV to the tumor cells and its internalization by endocytosis (Kalim et al., 2017). In our study, most tumor cells showed CD30 expression on the membranes. However, some cases exhibited CD30 in the cytoplasm only with absence of membrane expression. Given the clinical application of CD30 targeting, the variability of CD30 protein distribution between the cytoplasm and the cell membrane in RSCs, with focus on its potential manipulation, is important. The cytoplasmic expression of CD30 in the absence of membrane expression was observed previously (Schmidt et al., 1998; Diepstra et al., 2007; Weiss, 2013). This CD30 cytoplasmic expression pattern observed in some cases suggests an alteration in the regulation of membrane trafficking. Similar to cytoplasmic expression of MHCII in CHL (Diepstra et al., 2007) and in diffuse large B cell lymphoma (Kendrick et al., 2017), cytoplasmic CD30 expression in CHL may represent a distinct biology, as a possible response to signals from the microenvironment. The mechanisms of intracellular trafficking for membrane expression and/or degradation of CD30 are unknown, but a malfunction of either would result in a punctate cytoplasmic staining. CD30 that is sequestered in the cytoplasm eliminates the binding capacity of the tumor cell to BV, and thereby restricting efficient delivery of the drug into the tumor cell. Similarly, sequestration of MHCII in diffuse large B-cell lymphoma was shown to worsen progression free survival (Kendrick et al., 2017). Therefore, understanding the mechanism of membrane expression and cytoplasmic sequestration of CD30 is clinically relevant and needs further study.

Prior to the recent publication of guidelines for CD30 IHC testing (Gru et al., 2023), CD30 positivity was defined by universal strong reactivity in lymphomas, while reporting of variable expression levels were neglected. The current goal of these recommendations is to provide more detailed and specific information about CD30 expression in lymphomas. Achieving the best signal to noise ratios for optimal and comparable CD30 IHC results poses many challenges due to pre-analytical variables. Fixation and tissue processing have been held responsible for discrepancies between immunohistochemical studies in the past. This is further supported by studies showing that quantitative image analysis and multi-spectral imaging were able to detect CD30 expression in some cases that were found to be negative by immunohistochemistry, thereby revealing the limitations of immunohistochemistry to detect very low levels of CD30 (Horwitz et al., 2014; Kim et al., 2015). The key for detecting low expressing tumors is the use of control tissue with known CD30 expression including those with low expression levels.

As shown in this study, the use of low expressing cells or tissues as control reveals the failure of various antibody dilutions, lot numbers and staining protocols to detect low expression of CD30.

The guidelines recommend tonsils as a positive and negative control for CD30 using a protocol that is calibrated to provide a weak to moderate staining reaction of interfollicular activated B- and T-cells and activated B-cells primarily located in the rim of the germinal centers (Gru et al., 2023). However, a recent study showed extreme inter-tonsil differences in the number and staining intensity of CD30 positive cells in formalin and paraffin embedded tonsils (Steiniger et al., 2020). When using cryosections for comparison, this study found more weakly-stained, CD30-positive cells in some tonsils (Steiniger et al., 2020). This suggests that the cell array used in this study may be more optimal as control tissue. Furthermore, this study is in alignment with the guidelines, which recommend fixation in a 10% neutral-pH phosphate-buffered formalin for 8 to 72 hours, with a preferred 24-hour maximum. (Gru et al., 2023). Also, the clone BerH2 antibody used in this study, is the most common antibody used for routine assessment of CD30 expression in tissue specimens and provides good correlation between protein expression and mRNA levels (Bossard et al., 2014; Onaindia et al., 2016). Since CD30 expression can appear focally (Seliem et al., 2011) and most routine tumor specimens are obtained from biopsies, another limitation of CD30 detection could be the lack of a comprehensive analysis of the entire tumor bulk.

In summary, this study is an important step in attempting standardization of detection of CD30 expression. Further larger clinical trials are needed to determine whether CD30 expression levels as determined by IHC permit stratification of expected responses to anti-CD30 therapies.

## Figures and Tables

**Table 1.** This table details the case series, Hodgkin (HL) lymphoma sub-types, demographics and CD30 overall staining cores and staining patterns in tumor cells.

Case	Locators	HL Type	HL Subtype	Sex	Age	Stage	Extranodal Disease	CD30 pattern	Overall Score	Percent tumor cells positive Me, CP	Avenge Intensity Me*
1	Mediastinal mass	C	Nodular sclerosis	F	46	IA	absent	Me	7	100	3+
2	Lymph node	C	Nodular sclerosis	F	36	IIE	present	Me	7	100	3+
3*	Lymph node	C	Nodular sclerosis	M	48	IIX	present	Me	5	73	2+ *(3+,1+)
4	Mediastinal mass	C	Nodular sclerosis	F	36	-	-	Me	7	100	3+
5*	Lymph node	C	Nodular sclerosis	F	35	-	-	Me, CP	6	72, 17	2+ *(3+)
6	Lymph node	C,R	Nodular sclerosis	M	33	III	present	Me, CP	5	89, 3	1+
7	Supraclavicular mass	C	Nodular sclerosis	M	76	-	-	Me	7	96	3+
8*	Lymph node	C	Nodular sclerosis	F	29	II	present	CP	3	43	1+ *(3+)
9*	Lymph node	C	Nodular sclerosis	F	65	-	-	Me	3	37	1+ *(3+,2+)
10*	Neck mass	C	Nodular sclerosis	F	30	-	absent	Me	7	100	3+ *(2+,1+)
11	Lymph node	C	Nodular sclerosis	M	21	IIB	-	Me	7	100	3+
12*	Lymph node	C	Nodular sclerosis	M	14	I	absent	Me	7	79	3+ *(2+)
13*	Lymph node	C	Nodular sclerosis	M	26	-	-	Me, CP	6	89, 4	2+ *(1+,3+)
14	Lymph node	C	Nodular sclerosis	F	16	-	-	Me	7	76	3+
15	Liver	C	Nodular sclerosis	F	22	III	present	Me	7	93	3+
16	Lymph node	C	Nodular sclerosis	M	18	-	-	Me	6	63	3+
17*	Lymph node	C	Nodular sclerosis	M	16	II	present	Me	6	66	3+ *(2+,1+)
18	Lymph node	C	Nodular sclerosis	F	29	-	-	Me	6	57	3+
19	Lymph node	C	Nodular sclerosis	M	17	IVA	present	Me, CP	5	49, 1	3+
20*	Lymph node	C	Nodular sclerosis	M	24	-	-	Me, CP	6	56, 1	3+ *(2+)
21	Lymph node	C,R	Nodular sclerosis	M	22	-	-	Me	7	96	3+
22	Lymph node	C	Mixed cellularity	F	67	-	-	-	0	negative	0
23*	Neck mass	C	Mixed cellularity	M	21	-	-	Me, CP	4	57, 10	1+ *(2+)
24*	Neck mass	C	Mixed cellularity	M	19	IIIXA	present	Me	6	73	3+ *(2+)
25*	Lymph node	C	Mixed cellularity	F	67	IVA	present	Me, CP	5	72, 2	2+ *(3+,1+)
26	Lymph node	C	Lymphocyte rich	M	62	IIB	-	CP	3	33	1+
27	Left upper arm mass	C	Lymphocyte rich	F	57			Me	7	79	3+
28*	Lymph node	C	Lymphocyte rich	F	58	II	-	Me, CP	5	68, 4	2+ *(3+,1+)
29	Lymph node	C	Lymphocyte depleted	M	36	-	-	Me	6	57	3+
30	Lymph node	NC	NLP	F	51	-	-	-	0	negative	0
31	Lymph node	NC	NLP	M	35	-	-	-	0	negative	0
32*	Lymph node	NC	NLP	M	4	-	-	Me	4	49	2+ *(1+,3+)
33	Lymph node	NC	NLP	M	18	-	-	-	0	negative	0

Case series demographics, classification and subtypes, and CD30 staining patterns and scores for 33 cases of Hodgkin Lymphoma. Age, sex, and location were taken from original pathology reports. C, classical; NC NLPHL, NPL nodular lymphocyte predominant; R, recurrent; M, male; F, female; Me, membrane staining; CP, cytoplasmic punctate in the absence of membrane staining. ME, CP cases with mixed staining patterns in which tumor cells show either membrane staining (first number) or cytoplasmic staining in the absence of membrane staining (second number). Average intensity Me, average of intensity scores of membrane staining from three observation fields. In cases with intra-patient variability this value is the main intensity level expressed by the highest percentage of tumor cells. *Cases with intra-patient variability: the first intensity value in the brackets behind the average intensity shows the intensity level expressed by the second Iaege percentage of tumor cells (secondary intensity value). The second value in the brackets shows the intensity level expressed ty the lowest percentage of tumor cells (tertiary intensity value for membrane staining).

**Table 2.** Frequency of CD30 overall scores and staining patterns in HL sub-types. The overall scores were calculated from both the percentage of tumor cells that were positive and the staining intensity levels.

			Staining Patterns		
Histological Type	Expression levels (overall scores)	% of cases with overall scores	% of cases with Me only	% of cases with Me+CP	% of cases with CP only
Classical					
All types combined	High (6-7)	66 (19/29)	55 (16/29)	10 (3/29)	-
	Medium (4-5)	21 (6/29)	3 (1/29)	17 (5/29)	-
	Low (1-3)	10 (3/29)	3 (1/29)	-	7 (2/29)
	Negative (0)	3 (1/29)	-	-	-
Nodular sclerosis	High (6-7)	71 (15/21)	64 (14/21)	6 (1/21)	-
	Medium (4-5)	19 (4/21)	6 (1/21)	14 (3/21)	-
	Low (1-3)	10 (2/21)	6 (1/21)	-	5 (1/21)
Mixed cellularity	High (6-7)	25 (1/4)	25 (1/4)	-	-
	Medium (4-5)	50 (2/4)	25 (1/4)	25 (1/4)	-
	Negative (0)	25 (1/4)	-	-	-
Lymphocyte rich	High (6-7)	33 (1/3)	33 (1/3)	-	-
	Medium (4-5)	33 (1/3)	-	-	33 (1/3)
	Low (1-3)	33 (1/3)	33 (1/3)	-	-
Lymphocyte depleted	High (6-7)	100 (1/1)	-	100 (1/1)	-
Non-classical					
Nodular lymphocyte predominant	High (6-7)	0 (0/4)			
	Medium (4-5)	25 (1/4)	25 (1/4)	-	-
	Negative (0)	75 (3/4)			

Me, membrane staining only; CP, cytoplasmic staining only in the absence of membrane staining; Me+CP, both staining patterns present in tumor cells.

**Table 3.** Details how many RSC cells (percentage) per case express CD30 on the membrane. Staining intensity levels were disregarded here.

Scoring groups:			Percent of samples with only membranous staining pattern (Me)			
	Classical (N=29)					Nodular lymphocyte Predominant (NLPHL, N=4)
Percent of RSCs with membrane staining only	All types combined (N=29)	Nodular sclerosis (N=21)	Mixed cellularity (N=4)	Lymphocyte rich (N=3)	Lymphocyte depleted (N=1)	
100	17% (5/29)	24% (5/21)	0	0	0	0
75-96	28% (8/29)	33% (7/21)	0	33% (1/3)	0	0
50-74	38% (11/29)	29% (6/21)	75% (3/4)	33% (1/3)	100% (1/1)	0
25-50	14% (4/29)	14% (3/21)	0	33% (1/3)	0	25% (1/4)
5-24	0	0	0	0	0	0
<5	0	0	0	0	0	0
negative	3% (1/29)	0	25% (1/4)	0	0	75% (3/4)

**Table 4.** Intra-patient heterogeneity of intensity levels of CD30 staining on the membranes of RSCs, and the ratios of membrane (Me) and/or cytoplasmic (CP) staining patterns within same cases.

Case	Overall Score Staining pattern	% of all positive RSCs	Frequency of Intensity Levels of Membrane Staining (Me)					Cytoplasmic Staining (CP)	
			Total % RSCs with Me	Average Intensity for Me staining	% RSCs with Me 1+	% RSCs with Me 2+	% RSCs with Me 3+	% RSCs with CP staining	Average Intensity for CP staining
3	5, Me	73	73	2+	4	63	6	-	-
5	6, Me+CP	92	72	2+	0	50	22	17	2+
6**	5, Me+CP	92	89	1+	89	0	0	3	1+
9**	3, Me	37	37	1+	25	4	8	-	-
10	7, Me	100	100	3+	9	21	70	-	-
12	7, Me	79	79	3+	0	22	57	-	-
13	6, Me+CP	93	89	2+	37	34	18	4	2+
17	6, Me	66	66	3+	2	10	54	-	-
19	5, Me+CP	50	49	3+	0	0	49	3	3+
20	6, Me+CP	57	56	3+	0	8	48	1	2+
23**	4, Me+CP	67	57	1+	43	14	0	10	1+
24	6, Me	73	73	3+	0	35	38	-	-
25	5, Me+CP	74	72	2+	2	47	23	2	2+
28	5, Me+CP	72	68	2+	7	51	10	4	1+
32*	5, Me	49	49	2+	16	20	13	-	-

*: NLPHL

**: Low intensity levels that may have been missed with a less sensitive staining method. Me, membrane staining; CP, cytoplasmic punctate in the absence of membrane staining.
